# Supplementary material for: Effect of high-dose N-acetylcysteine on exacerbations and lung function in patients with mild-to-moderate COPD: a double-blind, parallel group, multicentre randomised clinical trial
Source: Nat Commun. 2024 Sep 30;15:8468. doi: 10.1038/s41467-024-51079-1 (PMC11442465; doi:10.1038/s41467-024-51079-1)
Supplement: Supplementary file 1 — Supplementary Information [file 41467_2024_51079_MOESM1_ESM.pdf]

SUPPLEMENTARY INFORMATION

Investigators of this study---2

Supplementary Table 1. Baseline characteristics of patients who underwent randomization. --6

Supplementary Table 2. Baseline characteristics of patients (full analysis set for lung function). --8

Supplementary Table 3. Baseline characteristics of patients who withdrew from the study. ---10

Supplementary Table 4. Medication compliance, concomitant drugs and smoking status during the study (full analysis set for exacerbation). -----------------------------12

Supplementary Table 5. Serum N-acetylcysteine levels in two group at baseline and 24 months in subgroup. ---13

Supplementary Table 6. Difference of CAT scores between the two groups at different time points. --14

Supplementary Table 7. Difference of mMRC scores between the two groups at different time points. --15

Supplementary Table 8. Comparison of medication-related adverse events between N-acetylcysteine and placebo groups (safety analysis set). -----------------------------16

Supplementary Figure 1. Kaplan-Meier plot of drop-out during the trial. ------------17

Supplementary Figure 2. Time to first acute exacerbation of COPD and hospitalization due to COPD (full analysis set for exacerbation). ---18

Investigators of this study

1. State Key Laboratory of Respiratory Disease, National Clinical Research Center for Respiratory Disease, National Center for Respiratory Medicine, Guangzhou Institute of Respiratory Health, The First Affiliated Hospital of Guangzhou Medical University.

   - Yumin Zhou M.D., Ph.D., Fan Wu, M.D., Heshen Tian, M.D., Ph.D., Zhishan Deng, M.D., Peiyu Huang, M.D., Sha Liu, M.D., Ph.D., Jieqi Peng, M.D., Zihui Wang, M.D., Longhui Tang, M.D., Jinzhen Zheng, M.D., Dongxing Zhao, M.D., Ph.D., Nanshan Zhong, M.D., Ph.D., Pixin Ran, M.D., Ph.D.

2. Shanghai Pudong Hospital of Fudan University

   - Yao Shen, M.D., Suizheng Cai, M.D., Jian Zhou M.D., Ph.D., Hong Xia M.D.

3. The Affiliated Zhongshan Hospital of Fudan University

   - Shujing Chen M.D., Ph.D.

4. Sir Runrun Shaw Hospital, Zhejiang University School of Medicine

- Yong Zhou M.D., Wenxia Zhou M.D.

5. The First Hospital of China Medical University

- Zanfeng Wang, M.D., Ph.D.

6. The First Hospital of Wenzhou Medical University

- Yanfan Chen, M.D., Xueding Cai, M.D.

7. Shenzhen People's Hospital

- Yingyun Fu, M.D., Yazhen Li, M.D.

8. The First Hospital of Guangxi Medical University

- Zhiyi He, M.D., Ph.D., Yanfei Bin, M.D.

9. Peking University Third Hospital

- Chun Chang, M.D., Ph.D.

10. The First Affiliated Hospital of Jinan University

- Shengming Liu, M.D., Ph.D., Li Chen, M.D.

11. The Second Xiangya Hospital of Central South University

- Ping Chen M.D., Ph.D., Shan Cai, M.D, Ph.D., Cong Liu, M.D., Fen Jiang, M.D.

12. Tongji Hospital of Tongji Medical College of Huazhong University of Science and Technology

- Huilan Zhang, M.D., Ph.D., Meijia Wang, M.D.

13. Tianjin Medical University General Hospital

- Jie Cao, M.D., Ph.D., Haiyan Zhao, M.D.

14. The Second Affiliated Hospital of Zhejiang University School of Medicine

- Yinghua Yin, M.D., Ph.D., Yichen He, M.D.

15. Ruijin Hospital, of Shanghai Jiao Tong University School of Medicine

- Qijian Cheng, M.D., Ph.D., Haiqin Zhang, M.D.

16. Lianping County People's Hospital

- Shuqing Yu, M.D., Lingshan Zeng, M.D., Xiangwen Luo, M.D., Jianhui Huang, M.D.

17. Wengyuan County People's Hospital

- Changli Yang, M.D.

18. The Second People's Hospital of Hunan Province

- Jianping Gui, M.D., Jia Tian, M.D.

19. Hunan Provincial People's Hospital

- Zhiguang Liu, M.D., Yongliang Jiang, M.D., Ph.D.

20. Huizhou First Hospital

- Zhe Shi, M.D., Yunzhen Zhang, M.D.

21. Guangzhou First People's Hospital

- Ziwen Zhao, M.D., Hua He, M.D.

22. The Third Affiliated Hospital of Guangzhou Medical University

- Liping Wei, M.D., Guoping Hu, M.D., Ph.D.

23. Liwan Hospital

- Fenglei Li, M.D.

24. Department of Respiratory and Critical Care Medicine, The Affiliated Hospital of Guangdong Medical University, The Second Affiliated Hospital of Guangdong Medical University.

    - Weimin Yao, M.D., Ph.D., Weiguang Lai, M.D., Hailin Zhou, M.D., Yuliang Cai, M.D., Yu Zhang, M.D.

— all in China.

Supplementary Table 1. Baseline characteristics of patients who underwent randomization.

**Characteristic**	**N-acetylcysteine Group** (N=484)	**Placebo Group** (N=484)	P Value
Age — year	62.5±8.4	62.6±8.0	0.79
Male sex — no. (%)	429 (88.6)	424 (87.6)	0.62
Body mass index — kg/m^2	22.7±3.6	22.9±3.4	0.57
Smoking status — no. (%)			0.13
Never smoked	79 (16.3)	90 (18.6)	
Former smoking	140 (28.9)	113 (23.3)	
Current smoking	265 (54.8)	281 (58.1)	
Smoking index — pack-year	44.9±26.8	44.1±23.9	0.72
Biomass use — no. (%)	259 (53.5)	267 (55.2)	0.61
Occupational exposure history — no. (%)	234 (48.3)	252 (52.1)	0.25
Family history of respiratory disease — no. (%)	87 (18.0)	76 (15.7)	0.35
Respiratory Medication for COPD — no. (%)	80 (16.5)	68 (14.0)	0.28
LAMA alone	40 (8.3)	36 (7.4)	0.63
ICS and LABA	28 (5.8)	25 (5.2)	0.67
Traditional Chinese medicine	7 (1.4)	10 (2.1)	0.46
Xanthine	9 (1.9)	3 (0.6)	0.08
Asmeton	3 (0.6)	3 (0.6)	1.00
LABA alone	5 (1.0)	0 (0.0)	0.06
LABA and LAMA	3 (0.6)	1 (0.2)	0.62
ICS alone	1 (0.2)	0 (0.0)	1.00
COPD exacerbations in the previous year — no. (%)	95 (19.6)	115 (23.8)	0.12
Spirometric values at baseline			
Before bronchodilator use			
FEV_1 — L	1.98±0.55	1.94±0.66	0.32
FEV_1 % of predicted value — %	78.3±16.7	77.8±17.1	0.64

FVC — L	3.32±0.79	3.26±0.76	0.26
FEV_1/FVC, %	59.6±7.5	59.3±7.9	0.67
After bronchodilator use			
FEV_1 — L	2.08±0.54	2.04±0.54	0.29
FEV_1 % of predicted value — %	82.5±15.8	81.8±16.8	0.55
FVC — L	3.41±0.75	3.38±0.74	0.49
FEV_1/FVC —%	60.7±7.5	60.2±7.7	0.32
Airflow reversibility — no. (%)	71 (14.7)	59 (12.2)	0.26
GOLD stage — no. (%)			0.08
Mild (stage 1)	274 (56.6)	247 (51.0)	
Moderate (stage 2)	210 (43.4)	237 (49.0)	
mMRC dyspnea scale score			
Mean score	0.82±0.80	0.82±0.78	0.97
Distribution — no. (%)			0.80
<2	406 (83.9)	403 (83.3)	
≥2	78 (16.1)	81 (16.7)	
CAT score	8.21±5.96	8.60±6.20	0.32

Data are n (%) or mean (SD). COPD, chronic obstructive pulmonary disease. ICS, inhaled corticosteroids. LABA, long-acting β-agonist. LAMA, long-acting muscarinic antagonist. FEV_1, forced expiratory volume in 1 second. FVC, forced vital capacity. GOLD=Global initiative for chronic obstructive lung disease. mMRC, modified Medical Research Council. CAT, chronic obstructive pulmonary disease assessment test.

Supplementary Table 2. Baseline characteristics of patients (full analysis set for lung function).

**Characteristic**	**N-acetylcysteine Group** (N=381)	**Placebo Group** (N=363)	P Value
Age — year	62.5±7.7	63.0±7.6	0.42
Male sex — no. (%)	348 (91.3)	325 (89.5)	0.40
Body mass index — kg/m^2	22.8±3.6	22.7±3.3	0.97
Smoking status — no. (%)			0.06
Never smoked	51 (13.4)	66 (18.2)	
Former smoking	112 (29.4)	84 (23.1)	
Current smoking	218 (57.2)	213 (58.7)	
Smoking index — pack-year			
Biomass use — no. (%)	215 (56.4)	216 (59.5)	0.40
Occupational exposure history — no. (%)	199 (52.2)	209 (57.6)	0.14
Family history of respiratory disease — no. (%)	67 (17.6)	61 (16.8)	0.78
Respiratory Medication for COPD — no. (%)	55 (14.4)	50 (13.8)	0.80
LAMA alone	27 (7.1)	24 (6.6)	0.89
ICS and LABA	19 (5.0)	18 (5.0)	0.99
Traditional Chinese medicine	6 (1.6)	10 (2.8)	0.27
Xanthine	6 (1.6)	2 (0.6)	0.29
Asmeton	3 (0.8)	3 (0.8)	1.00
LABA alone	4 (1.0)	0 (0.0)	0.12
LABA and LAMA	1 (0.3)	1 (0.3)	1.00
ICS alone	1 (0.3)	0 (0.0)	1.00
COPD exacerbations in the previous year — no. (%)	78 (20.5)	89 (24.5)	0.19
Spirometric values at baseline			
Before bronchodilator use			
FEV_1 — L	1.99±0.55	1.97±0.54	0.64
FEV_1 % of predicted value, %	78.1±16.7	78.7±17.5	0.62

FVC — L	3.35±0.77	3.32±0.72	0.61
FEV_1/FVC — %	59.1±7.4	58.9±8.0	0.73
After bronchodilator use			
FEV_1 — L	2.08±0.44	2.05±0.45	0.45
FEV_1 % of predicted value, %	82.3±16.1	82.8±16.9	0.71
FVC — L	3.44±0.73	3.43±0.70	0.84
FEV_1/FVC — %	60.4±7.6	60.0±7.8	0.40
Airflow reversibility — no. (%)	59 (15.5)	46 (12.7)	0.27
GOLD stage — no. (%)			0.59
Mild (stage 1)	209 (54.9)	192 (52.9)	
Moderate (stage 2)	172 (45.1)	171 (47.1)	
mMRC dyspnea scale score			
Mean score	0.80±0.76	0.80±0.76	0.90
Distribution — no. (%)			0.90
<2	322 (84.5)	308 (84.8)	
≥2	59 (15.5)	55 (15.2)	
CAT score	8.0±5.6	8.4±5.8	0.40

Data are n (%) or mean (SD). COPD, chronic obstructive pulmonary disease. ICS, inhaled corticosteroids. LABA, long-acting β-agonist. LAMA, long-acting muscarinic antagonist. FEV_1, forced expiratory volume in 1 second. FVC, forced vital capacity. GOLD=Global initiative for chronic obstructive lung disease. mMRC, modified Medical Research Council. CAT, chronic obstructive pulmonary disease assessment test.

Supplementary Table 3. Baseline characteristics of patients who withdrew from the study.

**Characteristic**	**N-acetylcysteine Group** (N=151)	**Placebo Group** (N=161)	P Value
Age —year	63.1±9.6	61.5±8.8	0.12
Male sex — no. (%)	124 (82.1)	129 (80.1)	0.65
Body mass index — kg/m^2	22.8±3.6	23.0±3.6	0.68
Smoking status — no. (%)			0.19
Never smoked	38 (25.2)	35 (21.7)	
Former smoking	42 (27.8)	34 (21.1)	
Current smoking	71 (47.0)	92 (57.1)	
Smoking index — pack-year	45.2±23.7	41.4±26.1	0.26
Biomass use — no. (%)	70 (46.4)	74 (46.0)	0.94
Occupational exposure history — no. (%)	60 (39.7)	65 (40.4)	0.91
Family history of respiratory disease — no. (%)	30 (19.9)	22 (13.7)	0.14
Respiratory Medication for COPD, no. (%)	32 (21.8)	21 (13.5)	0.06
LAMA alone	14 (9.3)	14 (8.7)	0.86
ICS and LABA	11 (7.3)	7 (4.3)	0.27
Traditional Chinese medicine	2 (0.6)	2 (0.6)	1.00
Xanthine	3 (2.0)	1 (0.6)	0.36
Asmeton	1 (0.7)	0 (0.0)	0.48
LABA alone	3 (2.0)	0 (0.0)	0.11
LABA and LAMA	3 (2.0)	0 (0.0)	0.11
ICS alone	0 (0.0)	0 (0.0)	1.00
COPD exacerbations in the previous year — no. (%)	23 (15.2)	40 (24.8)	0.035
Spirometric values at baseline			
Before bronchodilator use			
FEV_1 — L	1.93±0.55	1.86±0.53	0.26
FEV_1 % of predicted value — %	77.9±16.1	74.6±16.3	0.07

FVC — L	3.20±0.80	3.11±0.78	0.35
FEV_1/FVC — %	60.2±7.6	59.6±7.6	0.42
After bronchodilator use			
FEV_1 — L	2.00±0.50	1.95±0.52	0.39
FEV_1 % of predicted value —%	82.0±14.9	78.7±16.2	0.07
FVC — L	3.27±0.74	3.24±0.78	0.70
FEV_1/FVC — %	61.0±7.3	60.4±7.3	0.46
Airflow reversibility — no. (%)	19 (12.6)	23 (14.3)	0.66
GOLD stage — no. (%)			0.03
Mild (stage 1)	88 (58.3)	74 (46.0)	
Moderate (stage 2)	63 (41.7)	87 (54.0)	
mMRC dyspnea scale score			
Mean score	0.95±0.90	0.92±0.84	0.73
Distribution — no. (%)			0.60
<2	120 (79.5)	124 (77.0)	
≥2	31 (20.5)	37 (23.0)	
CAT score	8.9±6.9	8.8±7.2	0.92

Data are n (%) or mean (SD). COPD, chronic obstructive pulmonary disease. ICS, inhaled corticosteroids. LABA, long-acting β-agonist. LAMA, long-acting muscarinic antagonist. FEV_1, forced expiratory volume in 1 second. FVC, forced vital capacity. GOLD=Global initiative for chronic obstructive lung disease. mMRC, modified Medical Research Council. CAT, chronic obstructive pulmonary disease assessment test.

Supplementary Table 4. Medication compliance, concomitant drugs and smoking status during the study (full analysis set for exacerbation). *.

	**N-acetylcysteine Group** (N=464)	**Placebo Group** (N=460)	P Value
**Medication compliance** — no. (%)			0.13
Compliance ≥80%	329 (70.9)	330 (71.7)	
Compliance 50-80%	127 (27.4)	113 (24.6)	
Compliance < 50%	8 (1.7)	17 (3.7)	
**Respiratory medication** — no. (%)	112 (24.1)	107 (23.3)	0.75
Traditional Chinese medicine†	31 (6.7)	39 (8.5)	0.30
β2 agonists/inhaled corticosteroids†	30 (6.5)	30 (6.5)	0.97
Anticholinergic agents†	41 (8.8)	34 (7.4)	0.42
Xanthine†	21 (4.5)	14 (3.0)	0.24
β2 agonists†	14 (3.0)	16 (3.5)	0.69
Asmeton†	14 (3.0)	14 (3.0)	0.98
Montelukast sodium†	6 (1.3)	9 (2.0)	0.43
Inhaled corticosteroids†	4 (0.9)	2 (0.4)	0.69
β2 agonists/Anticholinergic agents†	3 (0.6)	1 (0.2)	0.62
**Smoking status**‡ — no. (%)			0.33
Never smokers	70 (15.1)	85 (18.5)	
Sustained smokers	218 (47.0)	224 (48.7)	
Intermittent smokers	52 (11.2)	46 (10.0)	
Stopped smoking	33 (7.1)	35 (7.6)	
Sustained ex-smokers	124 (26.7)	105 (22.8)	

* Data are shown as n (%).

† Used alone or in combination.

‡ Never smokers referred to subjects who were never smoking in his life; continuing smokers referred to subjects who were recorded as smoking at baseline and having continued to smoke at all visits; intermittent smokers referred to subjects who changed their smoking behavior from randomization on at least one visit; stopped smoking referred to subjects who were recorded as smoking at baseline and did not smoke during the trial; sustained ex-smokers referred to subjects who were recorded as having quit smoking prior to randomization and having maintained smoking abstinence at all visits. Chi-square or Fisher's exact test was adopted.

Supplementary Table 5. **Serum N-acetylcysteine levels in two group at baseline and 24 months in subgroup.**

N-acetylcysteine (ng/L)	N-acetylcysteine Group	Placebo Group	P Value
**Baseline**			
N	213	208	
Mean ± standard deviation	26.9±9.1	26.6±13.8	0.79
Median (interquartile range)	25.1 (20.9-29.9)	23.6 (19.9-30.1)	0.23
**Month 24**			
N	196	202	
Mean (standard deviation)	252.6±490.1	31.7±10.4	**<0.001**
Median (interquartile range)	142.0 (34.2-245.3)	29.1 (23.9-38.3)	**<0.001**

Data are shown as mean ± standard deviation or median (interquartile range).

Mixed-effects model for repeated measures is adopted.

Supplementary Table 6. Difference of CAT scores between the two groups at different time points.

**Follow-up**	**N-acetylcysteine**		**Placebo**		**Difference (Placebo - N-acetylcysteine) (95% CI)**	**Adjusted P Value**
Baseline	n=464	8.1±0.3	n=460	8.5±0.3	0.4 (-0.3 to 1.2)	0.27
Month 3	n=462	5.9±0.2	n=454	6.1±0.2	0.3 (-0.5 to 0.8)	0.43
Month 6	n=422	5.4±0.2	n=416	5.2±0.2	-0.1 (-0.8 to 0.5)	0.74
Month 9	n=405	4.7±0.2	n=399	4.9±0.2	0.1 (-0.5 to 0.7)	0.50
Month 12	n=385	4.8±0.2	n=371	5.0±0.2	0.2 (-0.4 to 0.9)	0.36
Month 15	n=362	4.2±0.2	n=352	4.1±0.2	-0.1 (-0.7 to 0.5)	0.72
Month 18	n=354	4.2±0.2	n=344	4.4±0.2	0.2 (-0.4 to 0.8)	0.26
Month 21	n=348	4.2±0.2	n=342	4.5±0.2	0.3 (-0.3 to 0.9)	0.22
Month 24	n=332	4.0±0.2	n=319	4.1±0.2	0.1 (-0.5 to 0.7)	0.49

*Data were shown as mean (SE).

Mixed-effects model for repeated measures is adopted. We adopted the measured value sat each visit as the dependent variable and the fixed effects includes treatment allocation, individual baseline value, baseline smoking status, age, sex, body mass index, COPD exacerbations in the previous year, COPD treatment at baseline, centre, follow-up, interactions between the treatment allocations and participating centers. Subjects served as the random effect. The follow-up was treated as the categorical variable. Values at visit 1 served as the baseline.

Supplementary Table 7. Difference of mMRC scores between the two groups at different time points.

Follow-up	N-acetylcysteine		Placebo		Difference (Placebo - N-acetylcysteine) (95% CI)	Adjusted P Value
Baseline	n=464	0.82±0.04	n=460	0.80±0.04	-0.02 (-0.12 to 0.09)	0.75
Month 3	n=462	0.67±0.03	n=454	0.66±0.03	-0.01 (-0.10 to 0.09)	0.64
Month 6	n=422	0.63±0.03	n=415	0.62±0.03	-0.000 (-0.09 to 0.09)	0.87
Month 9	n=405	0.55±0.03	n=399	0.54±0.03	-0.01 (-0.10 to 0.08)	0.74
Month 12	n=385	0.52±0.03	n=371	0.58±0.03	0.06 (-0.04 to 0.15)	0.74
Month 15	n=362	0.58±0.04	n=352	0.57±0.03	-0.003 (-0.10 to 0.90)	0.74
Month 18	n=354	0.57±0.03	n=344	0.63±0.04	0.60 (-0.04 to 0.16)	0.52
Month 21	n=346	0.61±0.04	n=34-	0.62±0.03	0.01 (-0.09 to 0.11)	0.94
Month 24	n=332	0.60±0.03	n=319	0.60±0.04	0.01 (-0.09 to 0.10)	0.72

*Data were shown as mean ± SE.

Cochran’s and Mantel-Haenszel test was adopted to compare differences between two group.

Supplementary Table 8. Comparison of medication-related adverse events between N-acetylcysteine and placebo groups (safety analysis set).

**Variables**	**N-acetylcysteine Group** (N =484)	**Placebo Group** (N =484)	**P value**
**Adverse event** (Reported by >1% of patients) — no. (%)	117 (25.2)	98 (21.1)	0.16
Epigastric discomfort	13 (2.7)	11 (2.3)	
Gastrointestinal pain	7 (1.4)	15 (3.0)	
Coronary heart disease	3 (0.6)	7 (1.4)	
Cerebral infarction	5 (1.0)	3 (0.6)	
Dizziness	4 (0.8)	5 (1.0)	
**Severe adverse event** — no. (%)	40 (8.6)	31 (6.7)	0.32
**Death** — no. (%)	3 (0.6)	5 (1.0)	0.72

Data were shown as n (%).

Chi-square or Fisher's exact test was adopted.

Supplementary Figure 1. Kaplan-Meier plot of drop-out during the trial.

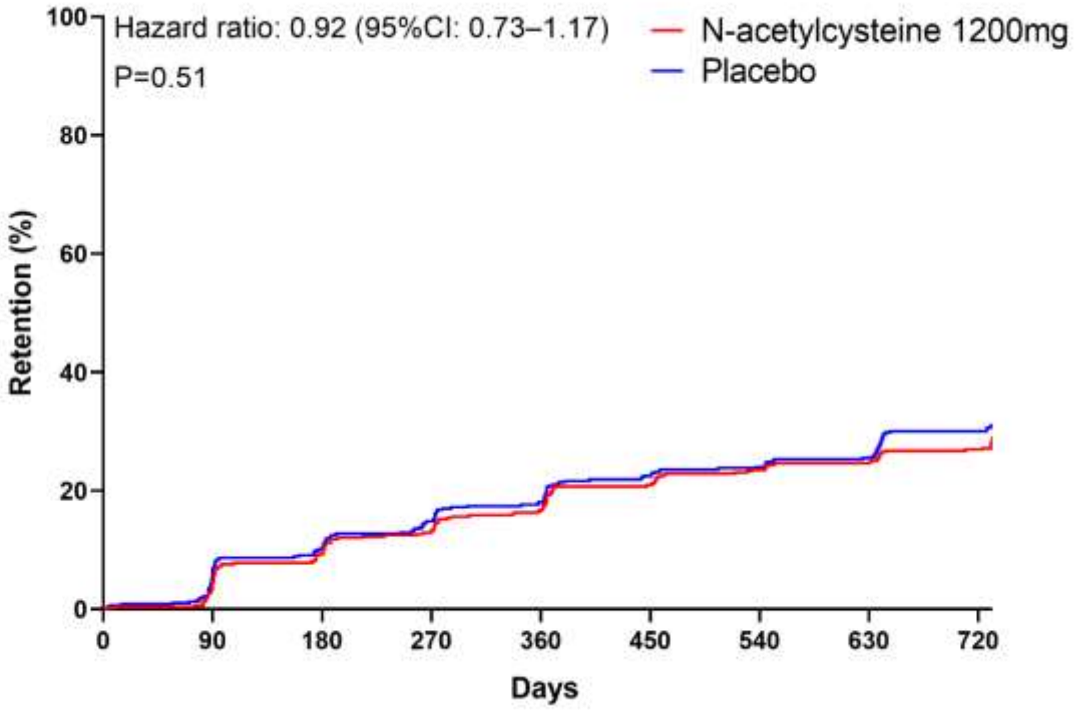

Cox proportional hazards regression model was adopted to compare differences between two group.

Supplementary Figure 2. Time to first acute exacerbation of chronic obstructive pulmonary disease (COPD) and hospitalization due to COPD (full analysis set for **exacerbation).**

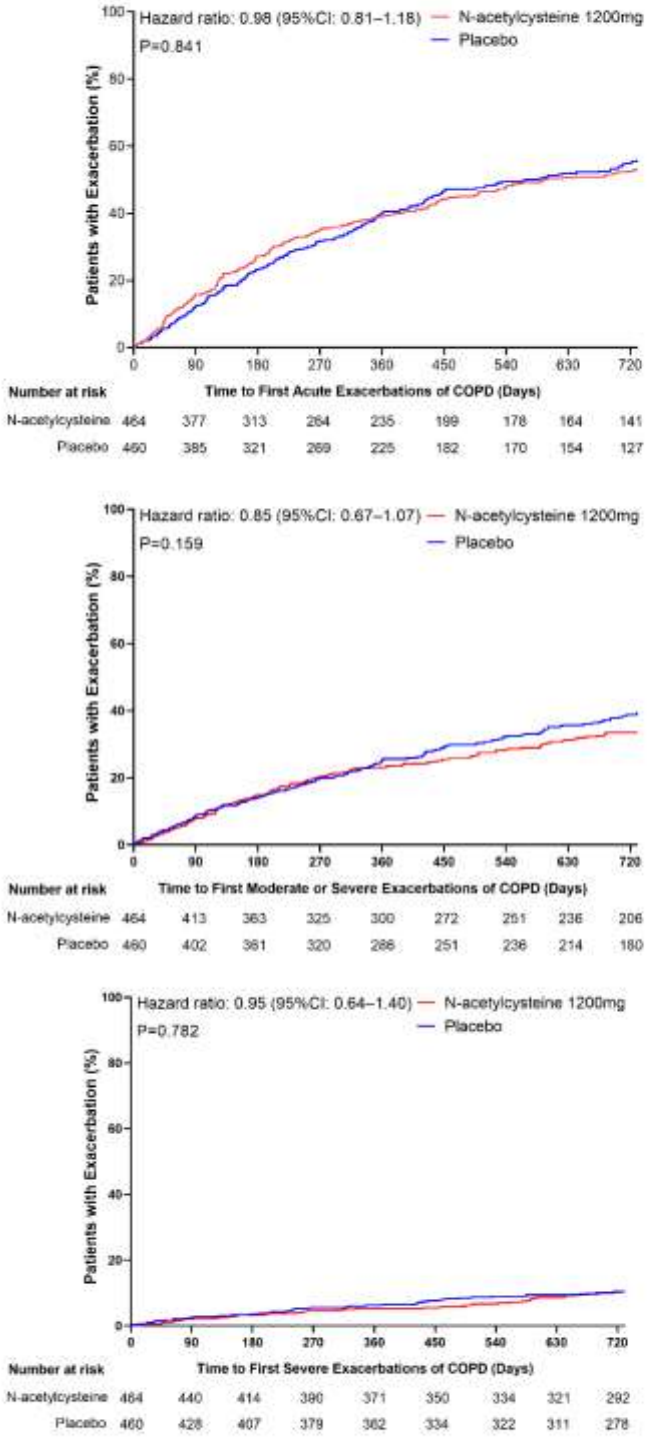

Cox proportional hazards regression model was adopted to compare differences between two group.
