# Supplementary material for: Effect of high-dose N-acetylcysteine on exacerbations and lung function in patients with mild-to-moderate COPD: a double-blind, parallel group, multicentre randomised clinical trial
Source: Nat Commun. 2024 Sep 30;15:8468. doi: 10.1038/s41467-024-51079-1 (PMC11442465; doi:10.1038/s41467-024-51079-1)
Supplement: Supplementary file 3 — Reporting Summary [file 41467_2024_51079_MOESM3_ESM.pdf]

nature portfolio

Corresponding author(s): Pixin Ran

Last updated by author(s): Jun 27, 2024

Reporting Summary

Nature Portfolio wishes to improve the reproducibility of the work that we publish. This form provides structure for consistency and transparency in reporting. For further information on Nature Portfolio policies, see our Editorial Policies and the Editorial Policy Checklist.

Statistics

For all statistical analyses, confirm that the following items are present in the figure legend, table legend, main text, or Methods section.

n/a	Confirmed	
☐	☒	The exact sample size (*n*) for each experimental group/condition, given as a discrete number and unit of measurement
☐	☒	A statement on whether measurements were taken from distinct samples or whether the same sample was measured repeatedly
☐	☒	The statistical test(s) used AND whether they are one- or two-sided<br>*Only common tests should be described solely by name; describe more complex techniques in the Methods section.*
☐	☒	A description of all covariates tested
☐	☒	A description of any assumptions or corrections, such as tests of normality and adjustment for multiple comparisons
☐	☒	A full description of the statistical parameters including central tendency (e.g. means) or other basic estimates (e.g. regression coefficient) AND variation (e.g. standard deviation) or associated estimates of uncertainty (e.g. confidence intervals)
☐	☒	For null hypothesis testing, the test statistic (e.g. *F*, *t*, *r*) with confidence intervals, effect sizes, degrees of freedom and *P* value noted<br>*Give P values as exact values whenever suitable.*
☒	☐	For Bayesian analysis, information on the choice of priors and Markov chain Monte Carlo settings
☒	☐	For hierarchical and complex designs, identification of the appropriate level for tests and full reporting of outcomes
☒	☐	Estimates of effect sizes (e.g. Cohen's *d*, Pearson's *r*), indicating how they were calculated

Our web collection on statistics for biologists contains articles on many of the points above.

Software and code

Policy information about availability of computer code

Data collection	We use electronic case report forms to collect data from clinical trials.

Data analysis	A predefined analysis of exacerbation rate was performed using the Poisson regression model, with adjustment for exposure to treatment doses, age, sex, body mass index, smoking status, COPD exacerbations in the previous year, COPD treatment at baseline, and centre. It was found that the number of acute exacerbations in this study had an over-dispersed distribution during data analysis, so we further adjusted the deviance over-dispersed in the Poisson regression model38-40. The rate of COPD exacerbations per patient per year was estimated using the Poisson regression model. Time to the first exacerbation was evaluated using the Cox proportional hazards regression model. A mixed-effects model for repeated measures was used to compare the differences in FEV1 and FVC before and after bronchodilator use at each visit between the two groups. The value measured at each visit was used as the dependent variable. The fixed effects in the model included the treatment method, patients' baseline lung function values, and follow-up visits (treated as categorical variables), and the random effects included the interaction of treatment with follow-up and centre. We used a random coefficient regression model to compare the differences in the annual decline in FEV1, FEV1 % of predicted value, FVC, FVC % of predicted value, and FEV1/FVC between the two groups. The model regression coefficient was used to represent the annual decline in the treatment effect over time, and the maximum-likelihood algorithm for imputing missing data. A mixed-effects model for repeated measures was used to compare the CAT score at each time point after treatment between the two groups. Cochran's and Mantel-Haenszel test was used to compare the change in the mMRC score. The number of adverse events for both groups was displayed. Except for using the maximum likelihood algorithm to estimate missing data in the random coefficient regression model, no imputation was performed for missing data in the remaining analyses.<br>Patients who underwent randomization were treated with at least one dose of study medication, and had available post-baseline data on exacerbation were included in the full analysis set (FAS) for exacerbation, and those who had available spirometry data at 12 months or 24 months were included in the FAS for lung function. All randomized patients were included in the safety analysis set. Statistical analyses were performed using SAS 9.4 (SAS Institute, USA), and all reported P values are two-sided.

For manuscripts utilizing custom algorithms or software that are central to the research but not yet described in published literature, software must be made available to editors and reviewers. We strongly encourage code deposition in a community repository (e.g. GitHub). See the Nature Portfolio guidelines for submitting code & software for further information.

Data

Policy information about availability of data

All manuscripts must include a data availability statement. This statement should provide the following information, where applicable:

- Accession codes, unique identifiers, or web links for publicly available datasets
- A description of any restrictions on data availability
- For clinical datasets or third party data, please ensure that the statement adheres to our policy

All the individual participant data collected during the trial (including the data dictionary) will be available, after deidentification, immediately after publication with no end date. Access to data related to this study was restricted due to concerns about patient privacy. Applications for access to data should be made in writing to Prof. Pixin Ran (pxran@gzhmu.edu.cn) who will arrange for request be reviewed, data will be provided within 1 month after approved by investigators and data transfer agreement has been signed.

Research involving human participants, their data, or biological material

Policy information about studies with human participants or human data. See also policy information about sex, gender (identity/presentation), and sexual orientation and race, ethnicity and racism.

Reporting on sex and gender	Regarding the use of the terms sex (biological attribute) and gender (shaped by social and cultural circumstances), we havecarefully checked in the entire manuscript and made corresponding revision in order to avoid confusing both terms.in addition, we guarantee that the manuscript meets the guideline requirement of the journal of Nature Medicine, includingguidance on Sex and Gender reporting.<br>1) We used sex to report the biological factors for male and female patients<br>2) Sex was not considered in the study design. Both male and female patients were eligible<br>3) The sex was summarized as part of demographic characteristics in Table 1. The sex of participants was collected accordingto the identity information provided by the patients. No individual-level data were reported.<br>4) No prior analvsis was sex-based. Post hoc analysis was performed to explore the effect of NAC subgrouped by sex.
Reporting on race, ethnicity, or other socially relevant groupings	This study was conducted in China and no specific race was selected during the subject recruitment phase. In the end, all participants included in this study were Chinese.
Population characteristics	Baseline characteristics were comparable between the two groups of patients both in randomization and in the FAS for exacerbation as well as for lung function (Table 1 and Extended Data Table 1-2). In the FAS for exacerbation, mean age of patients was 62.5 ± 8.4 years and 62.6 ± 8.0 years, mean FEV1 after bronchodilator use was 2.08 ± 0.54 L (82.4 % of the predicted FEV1) and 2.04 ± 0.54 L (82.0 % of the predicted FEV1) in the N-acetylcysteine and placebo groups, respectively (Table 1). Baseline characteristics of the patients who withdrew were comparable in the two groups (Extended Data Table 3). There was also no difference in medication compliance, smoking status, and respiratory medications for COPD between two groups at baseline or during the study (Table 1 and Extended Data Table 4). The peripheral blood N-acetylcysteine concentration in the N-acetylcysteine group was significantly higher than in the placebo group (Extended Data Table 5).
Recruitment	This is a multicentre, double-blind, parallel-group, placebo-controlled, randomized controlled trial at 24 centres in China from September 7, 2017 through January 3, 2022. We performed pulmonary function tests in the community to initially screen eligible subjects and recommended them for inclusion in this study. At the same time, this study also recruited subjects through research advertisements in the hospital. The representativeness of subjects with mild to moderate COPD recruited in this way is good. This effectively avoids the selection bias of recruiting subjects entirely from hospitals or from the community. 968 patients with mild-to-moderate COPD were randomized 1:1 to receive high-dose N-acetylcysteine (600 mg, twice daily) or placebo for two years. Eligible patients were 40-80 years old and GOLD stage 1–2 COPD (a ratio of forced expiratory volume in 1 second [FEV1] to forced vital capacity [FVC] ratio of <0.70 and an FEV1 of ≥ 50% predicted value after

	bronchodilator use), had chronic respiratory symptoms and/or risk factors for COPD (e.g., smoking, biofuel exposure, air pollution). The key exclusion criteria were (1) COPD exacerbation within four weeks prior to the screening period, (2) long-term treatment with N-acetylcysteine for more than three months prior to the screening period, (3) lung cancer, (4) asthma, (5) bronchiectasis, (6) interstitial lung disease, (7) post-pneumonectomy, (8) gastrointestinal ulcer, and (9) major systemic disease.
Ethics oversight	This trial was approved by the ethics committee of The First Affiliated Hospital of Guangzhou Medical University and each centre according to the requirements of Chinese clinical trial guidelines. All patients signed informed consent before enrolment.

Note that full information on the approval of the study protocol must also be provided in the manuscript.

Field-specific reporting

Please select the one below that is the best fit for your research. If you are not sure, read the appropriate sections before making your selection.

☒ Life sciences ☐ Behavioural & social sciences ☐ Ecological, evolutionary & environmental sciences

For a reference copy of the document with all sections, see nature.com/documents/nr-reporting-summary-flat.pdf

Life sciences study design

All studies must disclose on these points even when the disclosure is negative.

Sample size	The sample size was calculated based on the coprimary outcomes. First, referring to the Tie-COPD study and PANTHEON study9,15, we calculated the sample size using Poisson regression analysis to detect a difference of 0.23 per patient-year between the N-acetylcysteine group (0.27 per patient-year) and the placebo group (0.50 per patient-year) in the annual rate of exacerbations, with a two-sided significance level of 5% and a power of 90% based on an anticipated withdrawal rate of 35%. Thus, a minimum of 230 patients needed to be randomized per group. There are currently no studies on how much of a reduction in exacerbation rates would constitute a minimal clinically important difference37. Our hypothesized difference in exacerbation rate of 0.23 is equivalent to 46% fewer exacerbations in the treatment group compared with placebo. Therefore, we believe that a reduction of 0.23 per patient-year exacerbations is clinically significant, considering that previous clinical trials of COPD drugs were designed with a 15-25% reduction in exacerbation rate. Second, referring to the study by Pela and colleagues20, we calculated the sample size to detect the difference in FEV1 before bronchodilator use between the N-acetylcysteine group and the placebo group, assuming a difference of 100 ml and a standard deviation of 450 ml at month 24, with a two-sided significance level of 5% and a power of 80% based on an anticipated withdrawal rate of 35%. A minimum of 489 patients needed to be randomized per group. The sample size calculation for the selection of two coprimary outcomes was not adjusted for multiple comparisons. We chose the largest sample size calculated for the two coprimary outcomes for this study. Combining the calculation results of the two sample sizes, at least 489 patients needed to be randomized in each group. The statistical analysis plan, which was finalized before the data were finalized, specified the content of the statistical analysis in detail.
Data exclusions	The exclusion criteria were (1) COPD exacerbation within four weeks prior to the screening period; (2) long-term treatment with N-acetylcysteine for more than three months prior to the screening period; 3) patients with a clinical diagnosis of lung cancer, bronchiectasis, pneumoconiosis, asthma, interstitial lung disease, or other single restricted ventilation; 4) significant diseases other than COPD (A significant disease was defined as a disease or condition which, in the opinion of the investigator, may put the patient at risk because of participation in the study or may influence either the results of the study or the patients' ability to participate in the study); 5) patients with clinically significant abnormal baseline blood routine examination, blood biochemistry or urinary analysis if the abnormality defines a significant disease as defined in exclusion criteria No. 4; 6) Severe cardiovascular, neural, hepatic, renal, and hematologic diseases or malignancies that may interfere with the operation of the study; 7) Known moderate to severe renal impairment, judged by the investigator or creatinine clearance ≤50ml/min; 8) patients with phenylketonuria; 9) known hypersensitivity or intolerance to trial drugs; 10) patients with severe gastric ulcer or intestinal malabsorption; 11) patients with active pulmonary tuberculosis; 12) patients with life-threatening pulmonary embolism, α1-antitrypsin deficiency, or cystic fibrosis; 13) history of pneumonectomy; 14) pregnancy, lactation, or potential of pregnancy; 15) long-term oxygen therapy, frequent use of corticosteroids orally or intravenously (prednisone >10mg/d), or long-term use of antibiotics; 16) planned hospitalization or blood donation during the trial; 17) history of chronic alcohol or drug abuse, or any other conditions that may impact compliance; 18) involvement in other clinical studies at the same time; 19) patients who need long-term oxygen therapy and rehabilitation in the next 2 years.
Replication	N/A. This study the first randomized clinical trial to investigate the efficacy and safety of long-term with high-dose N-acetylcysteine in patients with GOLD stage 1–2 COPD.
Randomization	The randomization was centralized and stratified by centre, with block sizes of four.
Blinding	Investigators and centres maintain emergency sealed opaque envelopes for each numbered drug, containing the randomization code for a given patient, for emergency unblinding only in the event of a serious life-threatening adverse event. The principal investigator, physicians, patients, and statisticians were blinded to the drug allocation when the study was conducted. Zhejiang Jinhua Pharmaceutical (Hangzhou, China) manufactured and provided N-acetylcysteine and matched placebo. The matched placebo was identical to N-acetylcysteine in shape, colour, size, and packaging, but does not contain any active ingredients.

Reporting for specific materials, systems and methods

We require information from authors about some types of materials, experimental systems and methods used in many studies. Here, indicate whether each material, system or method listed is relevant to your study. If you are not sure if a list item applies to your research, read the appropriate section before selecting a response.

Materials & experimental systems

n/a	Involved in the study
☒	☐ Antibodies
☒	☐ Eukaryotic cell lines
☒	☐ Palaeontology and archaeology
☒	☐ Animals and other organisms
☐	☒ Clinical data
☒	☐ Dual use research of concern
☒	☐ Plants

Methods

n/a	Involved in the study
☒	☐ ChIP-seq
☒	☐ Flow cytometry
☒	☐ MRI-based neuroimaging

Clinical data

Policy information about clinical studies

All manuscripts should comply with the ICMJEguidelines for publication of clinical research and a completedCONSORT checklist must be included with all submissions.

Clinical trial registration

This study was registered on the Chinese Clinical Trial Registry, number ChiCTR-IIR-17012604.

Study protocol

The study design of this study was published and study protocol and statistical analysis plan was provided in Supplement material 【Tian, H. et al. High-dose N-acetylcysteine for long-term, regular treatment of early-stage chronic obstructive pulmonary disease (GOLD I-II): study protocol for a multicenter, double-blinded, parallel-group, randomized controlled trial in China. Trials 21, 780 (2020)】.

Data collection

This is a multicentre, double-blind, parallel-group, placebo-controlled, randomized controlled trial at 24 centres in China from September 7, 2017 through January 3, 2022. The screening period was one week, and clinical visits were then scheduled every three months. Patients were asked to record the management of exacerbation in a diary card and contact the investigator as soon as possible when respiratory symptoms appeared or worsened. COPD exacerbation was defined as the appearance or worsening of at least two major symptoms (cough, expectoration, purulent sputum, wheezing, or dyspnoea) persisting for at least 48 hours, following exclusion of cardiac insufficiency, pulmonary embolism, pneumothorax, pleural effusion, or cardiac arrhythmia has been ruled out. The severity of exacerbation was graded by the investigator according to the following categories: severe (requiring hospitalization); moderate (requiring outpatient or emergency department visits for increasing medication, including antibiotics and/or oral glucocorticoids); and mild (adding commonly used COPD medications at home). The mMRC dyspnoea scale and the CAT score were assessed at each visit.

Outcomes

The coprimary outcomes were the annual rate of total (mild, moderate, and severe) exacerbations and the difference in FEV1 before bronchodilator use at 24 months from baseline. The secondary outcomes included: the annual rate of moderate or severe exacerbations; the annual rate of severe exacerbations; the time to the first total exacerbation; the time to the first moderate-to-severe exacerbation; the time to the first severe exacerbation; the difference in FEV1 after bronchodilator use at 24 months from baseline; FVC before and after bronchodilator use at 24 months from baseline; annual decline in FEV1, FEV1 % of predicted value, FVC, FVC % of predicted value, and FEV1/FVC before and after bronchodilator use; mMRC score; CAT score; and adverse events. We performed prespecified subgroup analyses according to the mMRC score (<2 or ≥ 2), CAT score (<2 or ≥ 2), and GOLD stage (1 [mild] or 2 [moderate]). We also performed additional exploratory subgroup analyses according to smoking status (never smoker or ever smoker), use of medication for COPD at baseline (Yes or No), and COPD exacerbations in the previous year at baseline (Yes or No). All secondary, prespecified, and exploratory subgroup analyses should be interpreted as exploratory. These analyses have not been adjusted for multiple comparisons and are excluded from formal hypothesis testing.
